# Is Task-Irrelevant Learning Really Task-Irrelevant?

**DOI:** 10.1371/journal.pone.0003792

**Published:** 2008-11-24

**Authors:** Aaron R. Seitz, Takeo Watanabe

**Affiliations:** 1 Department of Psychology, University of California Riverside, Riverside, California, United States of America; 2 Department of Psychology, Boston University, Boston, Massachusetts, United States of America; James Cook University, Australia

## Abstract

In the present study we address the question of whether the learning of task-irrelevant stimuli found in the paradigm of task-irrelevant learning (TIPL) [1]–[9] is truly task irrelevant. To test the hypothesis that associations that are beneficial to task-performance may develop between the task-relevant and task-irrelevant stimuli, or the task-responses and the task-irrelevant stimuli, we designed a new procedure in which correlations between the presentation of task-irrelevant motion stimuli and the identity of task-targets or task-responses were manipulated. We found no evidence for associations developing between the learned (task-irrelevant) motion stimuli and the targets or responses to the letter identification task used during training. Furthermore, the conditions that had the greatest correlations between stimulus and response showed the least amount of TIPL. On the other hand, TIPL was found in conditions of greatest response uncertainty and with the greatest processing requirements for the task-relevant stimuli. This is in line with our previously published model that suggests that task-irrelevant stimuli benefit from the spill-over of learning signals that are released due to processing of task-relevant stimuli.

## Introduction

The phenomenon of task-irrelevant perceptual learning (TIPL) is one that has captured a growing interest in the field of perceptual learning. The basic phenomenon is that stimulus features that are irrelevant to a subject's task (i.e. convey no useful information to that task) can be learned due to their consistent presentation during task-performance. For example, Watanabe et al., [7] found that subjects showed sensitivity increases to a motion-direction stimulus after a prolonged exposure period to a subthreshold level of that motion-direction stimulus while the subjects performed a rapid serial visual presentation (RSVP) letter identification task. This finding has provided a challenge to attentional theories of perceptual learning [10]–[12] because the motion-direction stimulus was learned even though it provided no information regarding the identity, or temporal position in the sequence, of the targets of the RSVP task. A number of studies have reported similar findings [1]–[9] and confirmed that TIPL is a reliable phenomenon and have provided additional insights regarding the mechanisms underlying TIPL (for reviews see [1], [13]).

A leading theory is that TIPL occurs as a result of diffusely released learning signals that are triggered by performance of the RSVP task, which the subjects perform while being exposed to the task-irrelevant stimuli [1]. Evidence for this was provided by Seitz and Watanabe [4], where subjects were asked to perform a RSVP task in which correlations were introduced between the motion direction stimuli and the RSVP task targets. Specifically, a particular motion-direction was consistently paired with the task targets (such that it temporally enveloped the target letters) and other motion directions were temporally paired with the task distractors. After five daily sessions of this pairing procedure it was observed that an improvement of sensitivity had developed for the target-paired direction, but not for the other exposed directions. This result provided an important link between the subjects' task performance and the learning of the task-irrelevant motion stimulus.

However, the results of Seitz and Watanabe [4] raise a new question regarding whether the learning is truly task-irrelevant. For instance, if learning is based upon correlations with the “task-irrelevant” stimuli and behaviorally relevant events, then it might be the case that as the “task-irrelevant” stimuli are learned they in turn provide subjects with some benefit to the RSVP task-performance. The motion-direction stimuli used in Seitz and Watanabe [4] were regarded to be task-irrelevant because these stimuli were presented at subthreshold levels and they did not directly indicate the identity of the target letters. However, it is possible that subjects learned to use the paired-direction as a cue that indicated to the subjects that the target was about to appear.

This possible benefit that improved processing of the motion-direction stimuli may have for performance of the RSVP task seems particularly likely given the proposed similarity between the mechanisms underlying TIPL and those of reinforcement learning [1]. In Seitz and Watanabe [4], the paired-direction can be viewed as a predictor of the RSVP target and the same signals that result in learning of paired-direction would be well-placed to form an association between the paired-direction and the task-targets. Similarly, while in the Watanabe et al., [7] study only a single direction was presented during exposure, the motion sequence did indicate the start of the trial and could also cue subjects that the targets were about to appear. In fact, the duration of motion sequence in each trial of Watanabe et al., [7] was the same as that for each motion-direction presentation of Seitz and Watanabe [4] and thus the potentially task-relevant information (ie a temporal cuing effect) would be very similar between the two studies. Consistent with this idea performance on the RSVP tasks has been consistently reported to improve across the training sessions [2], [4], [8], but these studies were not designed to disentangle the potential contribution of the motion-stimuli to the RSVP task performance.

Here we directly tested the hypothesis that motion-direction stimuli, which were previously considered “task-irrelevant”, could in fact benefit subjects in their performance on the RSVP task. To explore this, we used a procedure very similar to that utilized by Watanabe et al., [7], however, here, we manipulated the correlational structure between the motion-direction stimuli and the targets and motor responses of the RSVP task. We examined both how the direction stimuli influenced performance on the RSVP task and also which conditions yield best learning of the motion-direction stimuli. We replicate the previously reported sensitivity benefits for the motion-direction stimuli, however, we find no evidence that this enhanced motion processing benefits RSVP task performance.

## Methods

### Participants

Seven subjects (4 male and 3 male, age range 18–25 years), who were naïve as to the purpose of the study, participated and received payment for their completion of the experiment. All subjects reported good ocular health and had a binocular (corrected) visual acuity (tested on-site) of 20/40 Snellen or better. Informed consent was obtained in writing from all the subjects and the experiments were conducted in accordance with the IRB approved by the Committee on Human Research of the Boston University and with the Declaration of Helsinki.

### Apparatus

The stimuli were presented using Psychophysics Toolbox [14], [15] for MATLAB (The MathWorks, Natick, MA) on a Macintosh G4 computer. The stimuli appeared on a ViewSonic VX922 19" monitor with resolution of 1280×1024 pixels and minimum response time of 2 ms and a refresh rate of 75hz. All experimental procedures were conducted under binocular viewing conditions and a chin rest was used to maintain the subject's head position. Subjects made responses using a computer mouse and keyboard.

### Motion-Direction Stimuli

A random dot kinematogram (RDK) motion stimulus [16] was employed with white dots (0.2° diameter) in a 1°–15° annulus with a dot density of 16.7 dots per deg^2^/s and dot speed of 12 deg/s and in which 3 dot-movies are interleaved. In this motion algorithm, the subset of coherently moving dots is newly chosen in each frame, and the probability of a given dot lasting more than one frame is the same as the coherence level; positions of non-coherent dots are randomly generated for each frame. For example, for the 5% coherent motion display, 5% of the dots in a successive frame will move in the same direction and speed (signal dots) while the remaining 95% will be replaced randomly (noise dots). The RDK stimuli were generated in real time so that each trial contained a unique motion stimulus. Because perception of cardinal directions may be robust to training [17], we employed a set of non-cardinal directions [10°, 70°, 130°, 190°, 250°, 310°] in this study.

### Procedure

**Figure 1:**
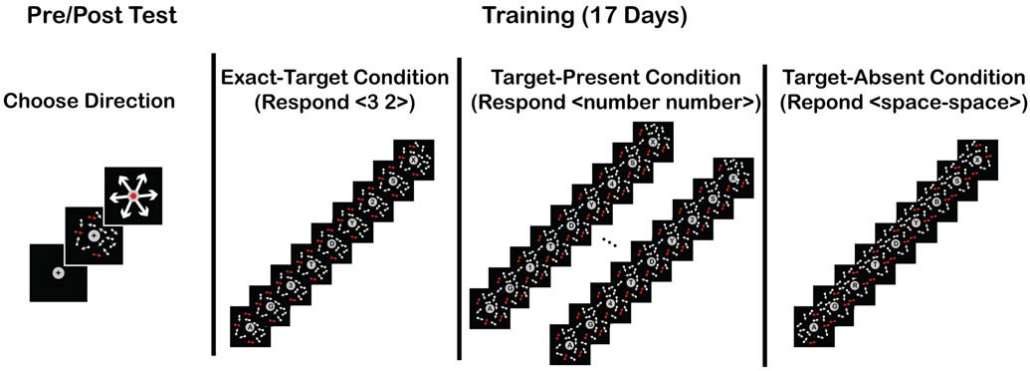
Design of Experiment. Tests, subjects conducted tests before and after training in which they reported the direction of motion coherence by selecting an arrow with a computer mouse. Training, a given trial was from one of three conditions; in the exact-target condition the targets were always the same for a given subject (here <3 2>), in the target-present trials the targets could be any number combination other than that presented in the exact-target condition, and in the target-absent condition no target was presented were to respond <space-space>. Red arrows indicate direction of coherence; a different direction of motion coherences was paired with each trial type.

The experiment consisted of twenty-one sessions; first a practice session to acquaint subjects with the motion-direction discrimination RSVP tasks, second a pre-test to measure sensitivity for various motion directions, then seventeen training sessions, and finally a post-test that was the same as the pre-test (see Figure 1 for schematics). Each session was conducted on a separate day with each subject conducting an average of five sessions per week.

Note, due to a computer glitch, data from the first 7 training days were irrecoverably corrupted in the first 4 subjects who were run. This problem was fixed and an additional 3 subjects were run to compensate for this problem. Accordingly, training data are reported for all 7 subjects for the final 10 days of training and for the 3 subjects for the full 17 days. We do not think that this affects the validity of the results because the most relevant data regard the relative performance between conditions at the end of the training period (for which we have data from all the subjects), and there were no notable effects in the first phase of training for the subjects for whom we have a complete data set.

### Pre/post Motion-Direction Sensitivity Tests

To measure sensitivity changes resulting from the training stage two test sessions were conducted, one before and one after the training stage. In contrast to the exposure (training) stage in which only 5% coherent motion was presented, in the test stages 5%, 15%, and 25% coherent motion were presented to each subject. In each trial, a fixation cross in the central circle was presented for 300-ms followed by the presentation of moving dots for 500-ms in a 2–15° annulus on an otherwise black screen. After another 300-ms six arrows appeared and subjects responded with a mouse click indicating which of the arrows was pointing in the direction of the just-viewed motion sequence. No feedback was given to reduce possible learning effects resulting from the testing sessions [18]. The order of presentation of the directions and coherence levels was pseudorandomly determined for each subject. Each test stage consisted of 6 directions×3 coherence levels (5%, 15%, 25%)×40 repetitions = 720 trials and took about 45 minutes to complete.

This 6-alternative-forced-choice (6AFC) procedure, using the method of constant stimuli, has been successfully used to indentify TIPL in previous studies [5], [19] and is similar to the 8AFC procedure used in the foundation studies of TIPL [4], [7]. In the present study, the six alternatives were chosen to accommodate the need to evaluate motion discrimination on the 3 directions exposed during training, which were equally spaced around the circle, and the 3 unexposed directions that were equally spaced from the exposed directions. In this and previous studies the exposed directions are counterbalanced across subjects so that biases specific to particular directions will average out across subjects.

### Training sessions

In the training sessions, subjects were asked to perform a foveal rapid serial visual presentation (RSVP) character identification task. Spatial configuration of the experiment is shown in Figure 1 and letter stimuli subtended ∼.75 degree of visual angle. In each trial, the RSVP sequence consisted of ten alphanumeric characters. Targets consisted of two numbers chosen from the set [‘1’, ‘2’, ‘3’, ‘4’] and distractors consisted of letter chosen form the set [‘A’, ‘B’, ‘C’, ‘D’, ‘E’, ‘F’, ‘G’, ‘H’, ‘J’, ‘K’, ‘L’, ‘M’, ‘N’, ‘P’, ‘R’, ‘T’, ‘W’, ‘X’, ‘Y’]. At the end of the trial, subjects had to type, in order of presentation, the identity of the two numeric targets or space-space (in target absent trials). No feedback was given. Each character in a sequence was presented for 35-ms and the interval between consecutive characters was 12-ms. The positions of the characters in a sequence were randomized for each trial with the constraint that the two targets could not appear consecutively.

For each subject, a set of 3 directions (with 120° spacing between each direction) was chosen from the set of six directions, which were tested. In the training phase, each of these directions was paired (with 83 1/3 % validity) with one of the following three trial types.

In exact-target trials, a pair of number targets was randomly selected for each subject with the constraint that two different numbers had to be presented (such as [1], [3], but not [1,1]). For each subject, one of the three motion directions that was selected for training was randomly assigned to be presented for the entire duration of exact-target trials.

In target-present trials, the target numbers were randomly selected on each trial with the exclusion of the set used in the exact-target condition. Thus, the motion-direction, randomly selected for each subject, which was exposed in target-present trials was 120° distant from the direction exposed in the exact-target trial.

In the target-absent trials, no targets were presented and instead the RSVP stream consisted of ten letters. Subjects were required to press the space-bar twice at the end of these trials. A third motion direction (different from those employed in the exact-target and target-present trials) was randomly assigned from the direction-set to be presented with target-absent trials with the constraint that it was 120° distant from the other assigned directions.

Each training session contained 1000 trials of the RSVP task with each condition represented with equal probability. In 25% of the trials (catch-trials) the regular direction pairing with that trial type was broken and one of the 3 paired directions was randomly chosen to be presented on that trial. In this way we were able to measure the impact that a particular direction had on performance for a given trial condition. All the analysis regard the data from these catch-trials.

### Analysis

**Figure 2:**
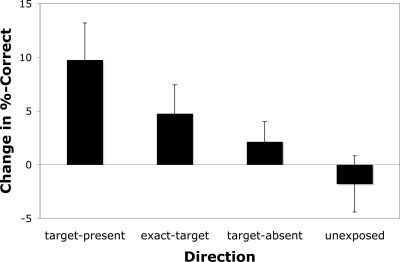
Performance change on the direction discrimination task, between pre- and post tests. Unexposed directions data represent the average across the 3 directions that were not exposed during the training phase. Error bars represent within-subject standard error [20]; see methods for details.

Data in Figure 2 is averaged across coherence levels for each direction in each session as has been done in previous studies of TIPL [5], [8].

**Figure 3:**
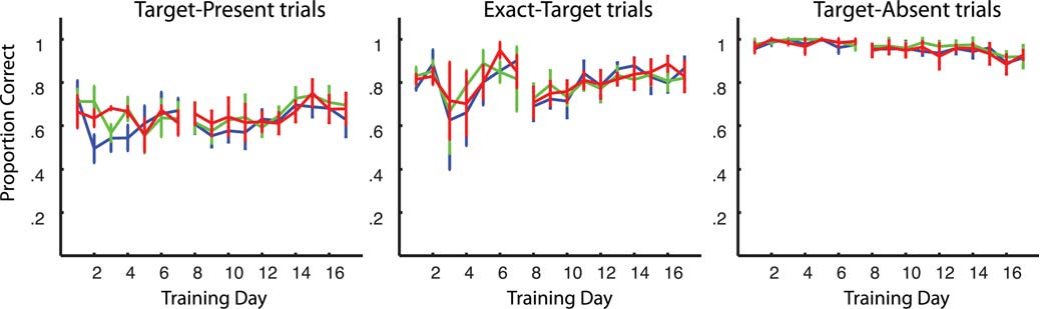
Results from the RSVP task performed during the training sessions. Plots represent data from target-present (left), exact-target (middle) and target-absent (right) trials, respectively. The break in the lines represents that the data from the first 7 days are from 3 subjects and that of last 10 days are from all 7 subjects. Each line represents trials in which a different direction was presented; exact-target direction (blue), target-present direction (green), target-absent direction (red). Error bars represent within-subject standard error [20]).

Error bars in Figures 2 and 3 represent within-subject standard error [20]. These are calculated by subtracting off the global mean of each subject for the testing or training sessions, respectively, from that subjects performance scores previous to the calculation of standard deviation across subjects. These error bars best reflect the use of paired t-tests, because variance in the relative differences, rather than the absolute difference, of performance in each condition is portrayed.

## Results

In this experiment, we manipulated the correlational structure between the motion-direction stimuli and the RSVP task in three different ways. In the “exact-target” condition a particular motion direction predicted precisely the identity of the target stimuli that would be presented in a particular trial, thus both predicting the visual stimulus and the motor-response. In the “target-present” condition a particular motion-direction predicted that targets would be present, but did not provide information regarding the identity of those targets (ie neither predicted the visual target-stimuli nor the motor response); this was the condition utilized in Watanabe et al., [7]. In the “target-absent” condition, a particular motion-direction predicted the absence of any target (thus predicted the motor response, but not the presentation of any particular visual stimulus). By introducing catch trials (where direction/target relations were randomized) into each session we could investigate the extent to which the motion-direction stimuli provided a benefit to subjects' performance of the RSVP task.

Given that our goal is to understand properties of TIPL, it is important to first demonstrate that the current study replicates the findings of improvements in direction-discrimination that have been previously reported. In Figure 2, we plot performance change between the direction tests and can see that directional learning did in fact occur. There was a significant improvement of relative performance of the target-present (t(6) = 2.5, p = .024; one-way paired t-test vs unexposed directions) and exact-target conditions (t(6) = 2.0, p = .045), but not for the target-absent condition (t(6) = 1.3, p = 0.12). While only the target-present showed significant learning effect compared to zero (t(6) = 2.6, p = .021) the comparison with the unexposed-directions is the more appropriate test because it accounts for baseline performance differences between the testing sessions. Also, psychometric (logistic) functions were fit on the data for each condition averaged across subjects using the psignifit toolbox version 2.5.6 for Matlab (see http://bootstrap-software.org/psignifit/), which implements the maximum-likelihood method described by Wichmann and Hill [21]. Thresholds changes followed the same pattern as found with performance data with a change of −4.2% coherence for the target-present condition, −3.8% coherence for the exact-target condition, −0.5% coherence for the target-absent condition, and .26% coherence for the unexposed directions. Notably, the target-present condition is most similar to the methods of Watanabe et al., [7] and here it is this condition that showed the most robust learning effect.

We next asked whether this directional-learning benefited the subjects' performance of the RSVP task. These Data are shown in Figure 3. Each plot represents performance on a different RSVP trial type; target-present (left), exact-target (middle) and target-absent (right). Looking across these plots it can be seen that subjects perform best on the target-absent trials, next best on the exact-target trials and worst on the target-present trials. This result was expected based upon the difficulty of target-identification in each condition, namely in the target-absent trials there is no target to identify, in the exact-target condition the target is always the same and in the target-present condition there is a set of different targets to which a choice in response must be made.

Looking within each of these plots we see that the three plotted performance curves are largely similar. Each curve (color) in these graphs represents the average performance across subjects on the catch trials in which a different direction than was normally paired with that trial type could be presented. The direction normally shown in the target-present condition is plotted in green, the direction paired with the exact-target condition in blue, and the direction paired in the target-absent condition in red; the color code for directions is the same in all three graphs. A repeated measures ANOVA confirms that there is no significant difference in performance between conditions in the target-present (F(2,12) = 1.93, p = 0.15), exact-target (F(2,12) = 0.18, p = 0.83) or target-absent (F(2,12) = 2.09, p = 0.13) conditions. There was some learning for the target-present (F(9, 54) = 2.14, p = .042) and the exact-target conditions (F(9,54) = 2.96, p = .0062) but not for the target-absent conditions (F(9,54) = 1.71, p = .11), which showed a little deterioration and was against ceiling. Notably, there was no interaction between training day and direction in any of the three conditions (F(18,108) = 0.66, p = 0.84, target-present; F(18,108) = 1.04, p = 0.42, exact-target; F(18,108) = 0.44, p = 0.98, target-absent). What is clear from the results is that the pairing of the direction stimuli with particular conditions of the RSVP task has an insignificant impact on the performance of the RSVP task. In short we failed to confirm the hypothesis that the motion-direction stimuli are task-relevant.

## Discussion

The results of this study support the model of task-irrelevant learning put forth by Seitz and Watanabe [1]. Learning is found for the motion-directions that were consistently paired with the trials of the RSVP task that contained targets (ie target-present and exact-target). This learning seems to be independent of a learned association between the motion-direction stimuli and the task-targets or with the subjects' response. While it is impossible to fully rule out that subtle associations were learned, or that they would develop with more training, it is clear that such associations, if any, are small and slow to develop.

An interesting finding in this study is that the directional learning seems to be more related to the difficulty of the RSVP task than the correlations between the paired-motion directions and the subjects' response or target-identity. Examination of Figure 2 shows the greatest learning effects for the target-present condition, an intermediate degree of learning for the exact-target, and poorest learning for the target-absent trials. Note that the target-present condition was the only condition in which the direction-stimuli failed to predict the correct response. In addition, in contrast to the exact-target condition, the motion-direction paired in the target-present trials didn't correlate exactly with the presentation of any particular visual stimulus. While the degree of learning did not seem to depend on the stimulus-stimulus or stimulus-response correlations, it did reflect the difficulty of each trial-type. The target-present trials were the most difficult and showed the greatest improvement in sensitivity for the motion-direction paired in these trials; the exact-target trials were a little easier and showed less directional learning; and the target-absent trials were the easiest and showed the least directional learning. While task-difficulty has been discussed often in relation to task-relevant learning these are the first results that indicate how task-difficult may shape task-irrelevant learning.

We have previously argued that task-irrelevant learning is due to a stimulus invariant learning signal and not a stimulus-directed attentional signal [1], [4], [7], [19]. This idea is further substantiated by the fact that learning was greatest in the most difficult condition. If learning was based upon the degree to which subjects could allocate attention to the motion stimuli, then one would predict the greatest learning in the easiest condition; where subjects could direct the most resources away from the task-relevant stimuli. The fact that the opposite result was found argues against this possibility. Likewise, one may predict that in the target-present trials more learning occurred because once the targets were found, subjects could release their attention from the letters and instead attend to the motion stimuli. However, in this case one would predict the greatest learning in the exact-target condition, in which, after learning the exact-target pair, subjects could release attention from the letters after only the first target was observed. However, while our results argue against these simple accounts of focused attention, we cannot rule out the possibility that attention plays are role in the observed learning effects (c.f. [1]).

Additionally, the fact that greatest learning was found for the motion-direction that was paired in the task-condition where performance was the lowest suggests that learning is not simply based upon correctly identifying the correct response. This rules out mechanisms that are simply dependant upon correct responses, since the most correct responses were made by subjects in the target-absent condition, which showed the least amount of learning for its paired direction. A possibility that we have previously hypothesized is that target-uncertainty is an important factor for TIPL [1], [22]. Since the target-present condition contained the largest set of response types, it is likely that in this condition reinforcement signals would be the greater, than in the other conditions, upon successful recognition of the targets. This is in contrast to the exact-target and target-absent conditions that entail unique responses, which each occur with a higher incidence than each of the responses types in the target-present trials. However, in addition to the degree of uncertainty, the target-present trials also required a greater level of processing of the RSVP stimuli in that subjects had to categorize all the characters as letters and numbers and process and maintain the identity of both numbers. In contrast, in the exact-target trials, the first target-number would predict with high probability the response for that trial and the target-absent trials in which there was no need to process the RSVP stimuli beyond the categorization between letters and numbers. To more directly determine what aspects of RSVP task-performance lead to TIPL, further research will be necessary which controls for overall performance while manipulating uncertainty and required levels of stimulus processing.

It is worth noting that no explicit feedback was given to subjects during performance of the RSVP task. Then where could the reinforcement come from? We have found that subjects have a high degree of confidence in their responses to the RSVP task and suggest that target-recognition asks as an internal reward [1], [4], [23]. Recent studies using liquid reinforcers have found similar task-irrelevant learning effects both in humans [24], [25] and monkeys [26], [27] and substantiate the role of reinforcement in task-irrelevant learning. While we currently don't have direct evidence of the underlying neural signals associated with TIPL, we have previously suggested neuromodulators such as acetylcholine, norepinephrine, and dopamine as candidate learning signals. Each of these neuromodulators are known to be involved in learning [28], [29] and have been proposed to have distinct roles as reinforcers [30]–[32]. Further research will be required to test whether these neuromodulators are involved in TIPL and, if so, what are their respective roles.

What use is TIPL if it doesn't provide any performance benefits to the subjects' task? We suggest that the brain has evolved mechanisms of learning and perception that work exceedingly well in most situations but are not always beneficial [2], [33]. We suggest that TIPL is a general mechanism that allows for the enhancement of stimulus processing for features that a consistently presented at behaviorally relevant times; in this case during target processing. As these stimuli become more relevant they can be brought in to the “awareness” of the individual and become accessible to attentional and decision processes that can more directly determine the “task-relevance” of the learned stimuli.

Our study addressed important questions regarding the learning that takes place in task-irrelevant learning. We find no evidence for associations developing between the learned (task-irrelevant) motion stimuli and the targets or responses to the subjects letter identification task. In fact the conditions that had the greatest correlations between stimulus and response showed the least amount of TIPL. On the other hand TIPL was found in conditions of greatest response uncertainty and with the greatest processing requirements for the task-relevant stimuli. This is in line with our previously published model that suggests that task-irrelevant stimuli benefit from the spill-over of learning signals that are released due to processing of task-relevant stimuli [13]. Future research will be required to understand more details of the properties of these learning signals
